# The independent association between vitamin B12 and insomnia in Chinese patients with type 2 diabetes mellitus: a cross-sectional study

**DOI:** 10.1038/s41387-022-00181-8

**Published:** 2022-01-17

**Authors:** Shuyuan Xiong, Zhiping Liu, Ning Yao, Xiaoru Zhang, Qian Ge

**Affiliations:** 1 Department of Endocrinology, the First Affiliated Hospital of Chongqing Medical University, 400016 Chongqing, China; 2 Chongqing Center for Disease Control and Prevention, 400016 Chongqing, China

**Keywords:** Nutrition disorders, Metabolism

## Abstract

**Background/objectives:**

Insomnia is highly prevalent in patients with type 2 diabetes mellitus (T2DM). This study therefore evaluated the associations between various micronutrients and insomnia in patients with T2DM.

**Subjects/methods:**

Between January 2018 and December 2020, a total of 418 T2DM patients with or without insomnia were recruited. Clinical and biochemical parameters, as well as micronutrient levels, were measured in each participant. Insomnia and sleep quality were assessed using the Athens Insomnia Scale and Pittsburgh Sleep Quality Index, respectively.

**Results:**

Insomnia was found in 24.16% of patients with T2DM. Compared with T2DM patients without insomnia, patients with insomnia had significantly higher levels of vitamin B12 (VitB12). Increased VitB12 was an independent risk factor for insomnia (OR 1.61 [1.06–2.45], *P* = 0.03). A cut-off value of 517.50 pg/ml VitB12 (*P* = 0.01, AUC 0.61, standard error 0.04) predicted insomnia risk. Moreover, increased VitB12 levels in patients with insomnia were closely correlated with the use of mecobalamin.

**Conclusions:**

This study suggests that elevated serum VitB12 level is independently associated with the incidence of insomnia and predicts increased insomnia risk in Chinese patients with T2DM.

## Introduction

Insomnia is highly prevalent among patients with type 2 diabetes mellitus (T2DM). A number of epidemiologic studies have revealed that insomnia is more common in patients with T2DM than in non-diabetic controls and independently associated with T2DM [1–5]. Long-term insomnia is not only a risk factor for the incidence of T2DM [6] but also worsens glycemic control [7] and increases the risk of dementia [8], depression [9], and cardiovascular diseases such as hypertension, myocardial infarction, and stroke in T2DM [10]. The mechanism by which diabetes may predispose to insomnia remains unclear. It has been proposed that pain from common complications such as peripheral neuropathy or nocturia from poor glycemic control may lead to disturbances in sleep [4, 11–13]. In addition, high blood glucose itself may have an adverse impact by altering neurotransmitter and autonomic functions [1]. Currently, treatment of insomnia in T2DM primarily involves symptomatic therapies, including relief of pain, adherence to good sleep hygiene, and use of hypnotics such as benzodiazepine receptor agonists and zolpidem. As the etiology of insomnia in diabetes is often multifactorial, a more complete understanding of associated risk factors is essential to better define its pathogenesis and develop effective management strategies.

Recent experimental studies demonstrated that micronutrients may impact the activity of presynaptic neurons or the synthesis of sleep-regulating neurotransmitters, including serotonin, glutamate, and melatonin [14]. However, the clinical relevance of micronutrients to sleep health has received less attention, and findings regarding some special micronutrients remain inconsistent. For example, several studies indicated a potential protective effect of 25-(OH) vitamin D (Vit D) on sleep [15–18]. In contrast, other studies reported an inverse relationship between Vit D and sleep quality [19, 20]. The association of vitamin B12 (VitB12) with sleep patterns is also not definitive. Some researchers found that VitB12 supplementation is associated with a decrease in sleep duration [15, 21, 22], whereas others found no significant association between VitB12 and sleep duration [23]. Moreover, some of these studies used dietary intake assessment methods to measure micronutrient status. This could have led to an inaccurate recall of individual dietary behaviors or measurement errors, which in turn could have biased the final measures of association. Furthermore, the effect of micronutrients on sleep has rarely been investigated in the population of T2DM patients treated with antidiabetes medications that can affect the absorption or supplementation of micronutrients. For these reasons, we investigated the association of serum levels of various micronutrients with insomnia among Chinese patients with T2DM. We hypothesized that some micronutrients might be independent contributors to insomnia in this population.

## Methods

### Study population

This was a cross-sectional study carried out between January 2018 and December 2020. A total of 418 participants who had T2DM were recruited among patients who visited the outpatient department clinics of the First Affiliated Hospital of Chongqing Medical University, Chongqing, China. The diagnosis of T2DM was based on the criteria of the World Health Organization (WHO 1999) [24]. All patients recruited were: (i) between 20 and 80 years of age; (ii) without diagnosed anxious depression; (iii) without diagnosed moderate-severe obstructive sleep apnea; (iv) without history of cerebral infarction/hemorrhage or other known nervous system disease; (v) non-alcohol abuser (alcohol consumption <140 g/week in men and <70 g/week in women [25]); (vi) scored <4 points on a numeric pain rating (0–10) scale. Exclusion criteria were patients who (i) had active infections; and (ii) had moderate or severe renal or hepatic dysfunction. All subjects recruited signed the consent form and agreed to take part in the present study. The research protocol was approved by the First Affiliated Hospital of Chongqing Medical University Ethical Committee (approval #2018-008).

### Sample size

The sample size was estimated using the following formula based on an unmatched case–control study design:

$${{{\mathrm{n}}}}1 = \frac{{(Z_{\alpha /2} + Z_{1 - \beta })^2\bar P(1 - \bar P)(r + 1)}}{{r(P1 - P2)^2}}{{{\mathrm{,}}}}\;{{{\mathrm{n}}}}2 = {{{\mathrm{rn}}}}1,\bar P = \frac{{P1 + rP2}}{{r + 1}};$$

The sample size was calculated using a 3:1 ratio of controls to cases according to the previously reported prevalence of insomnia in T2DM patients [2]. In the above formula, n1 represented the number of cases (T2DM with insomnia), and n2 represented the number of controls (T2DM without insomnia); P1 and P2 were estimated using the previously reported proportion of patients with Vit D within the normal range in the control and case groups, respectively (assuming the lowest odds ratio to be detected was 2.2) [18]. For the two-sided test, *α* was set at 0.05, and power was set at 80% (*β* = 0.2). The sample size estimated was at least 297 for the control group and 99 for the case group.

### Clinical and biochemical measurements

Data regarding sociodemographic parameters including lifestyle, drinking, smoking, past medical history, use of dietary supplements, and sleep were collected via face-to-face interviews, whereas the use of mecobalamin was determined via calling back interviews by two trained diabetes nurses. These nurses also performed the anthropometric measurements. Body weight and height were measured with the participants wearing light clothes and in bare feet to the nearest 0.1 kg and 0.1 cm, respectively. Body mass index (BMI) was calculated as the ratio between weight in kilograms and the square of height in meters (kg/m^2^). Blood pressure (BP) was measured on the nondominant arm in a seated position after a 10-min rest using an electronic BP monitor (OMRON BP-203RVIIIC, Matsusaka, Japan).

Blood was drawn after an overnight fast, and biochemical parameters were assessed using standard laboratory methods (for the sensitivity of detection methods, see Supplementary Table) at the Central Laboratory and Endocrine Laboratory of our hospital. C-peptide (C-P) was measured via electrochemiluminescence (Cobas e601, Roche, Germany). Hemoglobin A1c (HbA_1_c) was estimated by high-pressure liquid chromatography using a Premier HB-9210 analyzer (Primus Corp., USA). Serum lipids were measured as follows: total cholesterol using the cholesterol oxidase-HDAOS method (Wako, Osaka, Japan); triglycerides using the GPO-HDAOS glycerol blanking method (Wako); high-density-lipoprotein cholesterol using the immunoinhibition (direct) method (Wako), and low-density-lipoprotein cholesterol using the selective protection enzymatic (direct) method (Wako). Glutamic-pyruvictransaminase, glutamicoxalacetic transaminase, creatinine, and uric acid were measured using enzymatic methods on an autoanalyzer (HITACHI 7180, Ichige, Japan). Hemoglobin (Hb) content was measured using an automatic blood cell counter hematology analyzer (SysmexXE-5000, Japan). Potassium (K) and iron (Fe) were measured using an indirect choice electrode method and Ferrizine colorimetric method (Cobas 8000 c701, Roche, Switzerland), respectively. Magnesium (Mg), phosphorous (P), calcium (Ca), and sodium (Na) were measured using colorimetric methods (Cobas, 8000 c701, Roche, Switzerland). VitB12, folic acid, FT3, FT4, TSH, and cortisol were determined using an electro-chemiluminescence method (Unicel DxI 800 Immunoassay System, Beckman Coulter, USA). ACTH was determined using an automatic immunoanalyzer (Cobas e601, Roche, Switzerland). Vit D was measured using a chemiluminescence method (Liaison XL, DiaSorin, Italy).

### Evaluation of insomnia and sleep quality

Subjective insomnia was evaluated using the Chinese version of the Athens Insomnia Scale, consisting of 8 items (AIS-8). Each item was scored from 0 points (no problem) to 3 points (very severe problem). A summed AIS score of ≥6 was defined as insomnia [26, 27]. The diagnostic accuracy of the AIS has been validated in Chinese adults with a sensitivity of 91% and specificity of 87% [28].

Subjective sleep quality was assessed using the validated Chinese version of the Pittsburgh Sleep Quality Index (PSQI) [29]. The PSQI consists of 19 questions, generating 7 component scores (C1: sleep quality; C2: sleep onset latency; C3: sleep duration; C4: sleep efficiency; C5: sleep disturbance; C6: use of sleep medications; and C7: daytime dysfunction). Each component was scored on a 0- to 3-point scale. The scores of the seven components were then summed to yield a global PSQI score (range: 0-21), with higher scores indicative of worse sleep quality. The Chinese version of the PSQI with a score of >5 yielded a sensitivity and specificity of 98% and 55%, respectively, for identifying poor sleep quality [29].

### Definition of diabetic peripheral neuropathy

The clinical diagnosis of diabetic peripheral neuropathy (DPN) was determined using a questionnaire (Neuropathy Symptom Score) and physical examination (Neuropathy Deficit Score), which were performed by an experienced neurologist on all patients, as described previously [30]. The questionnaire consisted of four questions: positive responses were scored as 1–3 points according to an abnormal degree, and negative responses were scored as 0 points (see Table 1). A score of 3–4 points was considered mild symptoms, 5–6 points as moderate symptoms, and 7–9 points as severe symptoms. The physical examination included: (i) ankle reflexes, which were scored as normal (0 point), present only with reinforcement (1 point), or totally absent (2 points); (ii) vibration sense on the dorsum of the big toe (128-Hz tuning fork), pinprick sensation, thermal (cold/hot) discrimination, and pressure sensation, all of which were scored as normal (0 points) or reduced/absent (1 point). Likewise, a score of 3–5 points was regarded as a mild sign, 6–8 points as a moderate sign, and 9–12 points as a severe sign. Once either of the following conditions appeared ([i] moderate or severe signs; [ii] mild signs with moderate or severe symptoms), the diagnosis of DPN was established [30].

**Table 1:** General characteristics of subjects.

	T2DM without insomnia(*n* = 317)	T2DM with insomnia(*n* = 101)	*P*-value
Age (year)	58.33 ± 13.33	62.36 ± 11.80	**<0.01**
Female, *n* (%)	125 (39.43)	52 (51.49)	**0.03**
Duration (month)	108 (24–180)	120 (60–192)	**0.04**
Drinker, *n* (%)	111 (35.02)	24 (23.76)	**0.04**
Smoker, *n* (%)	141 (44.48)	35 (34.65)	0.08
BMI (kg/m^2^)	24.77 ± 3.84	24.40 ± 4.54	0.18
SBP (mmHg)	136.27 ± 20.03	135.85 ± 22.40	0.81
DBP (mmHg)	79.20 ± 12.00	77.82 ± 13.33	0.27
HbA_1_C (%)	9.64 ± 2.38	9.12 ± 2.52	0.09
F-CP (ng/ml)	1.82 (1.19–3.22)	2.02 (1.51–3.25)	0.39
TC (mmol/L)	4.43 ± 1.33	4.26 ± 1.55	0.16
TG (mmol/L)	1.24 (1.05–1.59)	1.17 (0.97–1.58)	0.20
LDL (mmol/L)	2.62 ± 1.07	2.39 ± 0.98	0.09
HDL (mmol/L)	1.07 ± 0.37	1.15 ± 0.39	**0.02**
ALT (IU/L)	18 (13–33)	20 (14–28)	0.69
AST (IU/L)	16 (13–23)	18 (14–22)	0.35
Cr (μmol/L)	64.50 (53.00–80.25)	66.00 (50.00–86.00)	0.60
UA (μmol/L)	336.68 ± 100.78	308.86 ± 95.08	**0.02**
Hb (g)	135.85 ± 20.03	131.66 ± 19.32	**0.02**
FT3 (pg/ml)	3.13 ± 0.49	3.11 ± 0.42	0.69
FT4 (ng/dl)	0.98 ± 0.16	0.98 ± 0.16	0.82
TSH (μIU/ml)	1.94 (1.27–2.80)	2.23 (1.35–3.45)	0.05
Cortisol (nmol/L)	355.10 ± 119.22	360.56 ± 138.52	0.71
ACTH (pg/ml)	33.21 ± 18.18	35.47 ± 21.90	0.58
Dietary supplement, *n* (%)			
Vitamin B complex	4 (1.26)	2 (1.98)	0.96
Vitamin D	11 (3.47)	2 (1.98)	0.67
Calcium	13 (4.10)	3 (2.97)	0.82
Multivitamin formula with minerals	12 (3.79)	8 (7.92)	0.09
Mecobalamin	45 (14.10)	25 (25.00)	**0.03**
DPN, *n* (%)	152 (47.95)	59 (58.42)	0.09
Use of Metformin, *n* (%)	201 (63.41)	60 (59.40)	0.47
Use of Insulin, *n* (%)	163 (51.42)	57 (56.44)	0.38
PSQI summed score	4.69 ± 2.24	12.01 ± 2.99	**<0.01**
C1 (sleep quality)	0.92 ± 0.55	2.06 ± 0.56	**<0.01**
C2 (sleep-onset latency)	0.79 ± 0.90	2.38 ± 0.93	**<0.01**
C3 (sleep duration)	0.46 ± 0.68	1.89 ± 0.99	**<0.01**
C4 (sleep efficiency)	0.21 ± 0.52	1.85 ± 1.04	**<0.01**
C5 (sleep disturbance)	1.04 ± 0.32	1.50 ± 0.52	**<0.01**
C6 (medications)	0.06 ± 0.40	0.21 ± 0.73	**0.01**
C7 (daytime dysfunction)	1.21 ± 0.91	2.12 ± 0.90	**<0.01**

Data are means ± SD or median (interquartile ranges) or number (percentage) for the indicated number of patients in each group. *P* values for comparisons between groups are based on unpaired *t*-test or Mann–Whitney Test (when the data are not normally distributed) or *χ*^2^ test. Bold entries represent significant *p* values. The *P* < 0.05 is considered to be significant.

*BMI* body mass index, *SBP* systolic blood pressure, *DBP* diastolic blood pressure, *FC-P* fasting C-peptide, *TC* total cholesterol, *TG* triglycerides, *HDL* high-density lipoprotein cholesterol, *LDL* low-density lipoprotein cholesterol, *ALT* alanine aminotransferase, *AST* aspartate aminotransferase, *Cr* creatinine, *UA* uric acids, *Hb* hemoglobin, *DPN* diabetic peripheral neuropathy, *PSQI* Pittsburgh Sleep Quality Index.

### Statistical analyses

Statistical analyses were performed using SAS 9.13 (SAS Institute, Cary, NC). Variables are presented as mean ± SD. Means of continuous variables were compared using the unpaired *t*-test or Mann–Whitney test (when the data were not normally distributed). Percentage differences between groups were compared using the *χ*^*2*^ test. Correlations were evaluated by Spearman analysis. Logistic regression analysis was performed to evaluate the independent risk factors associated with the presence of insomnia. Receiver operating characteristic (ROC) curve analysis was performed to evaluate the predictive efficiency and cut-off values of VitB12 for insomnia. Differences between groups were considered statistically significant at *P* < 0.05. For logistic regression analysis and ROC curve analysis, statistical significance was set at *P* < 0.05.

## Results

### Clinical and biochemical characteristics of subjects

Insomnia was observed in 24.16% of patients with T2DM in our study. As shown in Table 1, compared with the patients without insomnia, the patients with insomnia were older, had a higher proportion of females, a lower proportion of alcohol drinkers, and a longer duration of diabetes. Moreover, the patients with insomnia had lower Hb levels. There were no significant differences in BMI, BP, glycemic control (HbA_1_c), β-cell function (fasting C-peptide), pituitary-thyroid hormones, pituitary-adrenal hormones, presence of DPN, or medication usage (metformin and insulin) between the two groups. There was a small proportion of patients who took dietary supplements in at least one recent year. The proportion of supplement intake was similar between the two groups, except that mecobalamin (an active form of VitB12) was used more commonly in the insomniac group. As expected, the subjects with insomnia exhibited much worse sleep quality based on both the global PSQI score and on each subscale score.

### The association of micronutrient VitB12 with insomnia

We assessed the serum levels of Vit D, folic acid, VitB12, Ca, Mg, P, K, Na, and Fe in each participant. Only VitB12 levels were significantly higher in patients with insomnia (Table 2). Taking into account that age, alcohol use, gender, duration of diabetes, and Hb level (which have been shown to interfere with sleep quality [31]) differed between the groups, we performed a logistic regression analysis to confirm increased VitB12 as an independent risk factor for insomnia or poor sleep quality. In univariate regression analysis (no adjustment), serum VitB12 level exhibited an almost significant association with insomnia (OR: 1.50, 95% CI: 1.00–2.14; *P* = 0.05) but no association with poor sleep quality (global PSQI score>5) (OR: 1.28, 95% CI: 0.92–1.79; *P* = 0.14). Interestingly, after adjustment for the aforementioned confounders in model 1, VitB12 was significantly associated with the presence of insomnia (*P* = 0.02), conferring a 1.6-fold increase in the odds ratio for insomnia. Although DPN did not differ between the two groups, this complication of diabetes is often regarded as a sleep disruptor [11, 32]. Thus, we further adjusted for DPN in model 2. The associations between VitB12 and the presence of insomnia remained significant (*P* = 0.03; Table 3).

**Table 2:** The micronutrients in T2DM patients with or without insomnia.

	T2DM without insomnia(*n* = 317)	T2DM with insomnia(*n* = 101)	Reference ranges	*P*-value
Vitamin D (ng/ml)	18.19 ± 7.11	19.12 ± 7.94	30–100	0.19
Folic acid (ng/ml)	11.55 (8.60–15.73)	12.40 (9.60–17.50)	3.1–19.9	0.05
**VitB12 (pg/ml)**	**422.50 (293.00**–**626.75)**	**532.00 (325.00**–**726.00)**	**180**–**914**	**0.03**
Calcium (mmol/L)	2.32 ± 0.12	2.30 ± 0.13	2.10–2.55	0.48
Magnesium (mmol/L)	0.79 ± 0.08	0.79 ± 0.11	0.7–1.0	0.40
Phosphorus(mmol/L)	1.21 ± 0.18	1.22 ± 0.20	0.81–1.45	0.75
Potassium(mmol/L)	4.20 ± 0.40	4.19 ± 0.45	3.5–5.1	0.74
Sodium (mmol/L)	138.25 ± 3.50	137.58 ± 3.88	137–145	0.25
Iron (μmol/L)	14.17 ± 6.06	16.01 ± 6.08	7.8–32.2	0.50

Data are means ± SD or median (interquartile ranges) for indicated number of patients in each group. *P* values for comparisons between groups are based on unpaired *t* test or Mann–Whitney Test (when the data are not normally distributed). Bold entries represent significant *p* values. The *P* < 0.05 is considered to be significant.

Bold entries represent significant *p* values.

**Table 3:** Associations between the VitB12 and the presence of insomnia in T2DM patients in different Logistic Regression Models.

	OR (95% CI)	*P*-value
*No adjustment*		
VitB12	1.50 (1.00–2.14)	0.05
*Model 1: adjusted for age, sex, duration, drinker, hemoglobin*		
VitB12	1.62 (1.07–2.46)	0.02
*Model 2: further adjusted for the presence of DPN*		
VitB12	1.61 (1.06–2.45)	0.03

VitB12 data are natural logarithm transformed. The statistical significance is set as *P* < 0.05.

*OR* odds ratio, *CI* confidence interval, *DPN* diabetic peripheral neuropathy.

**Fig. 1:**
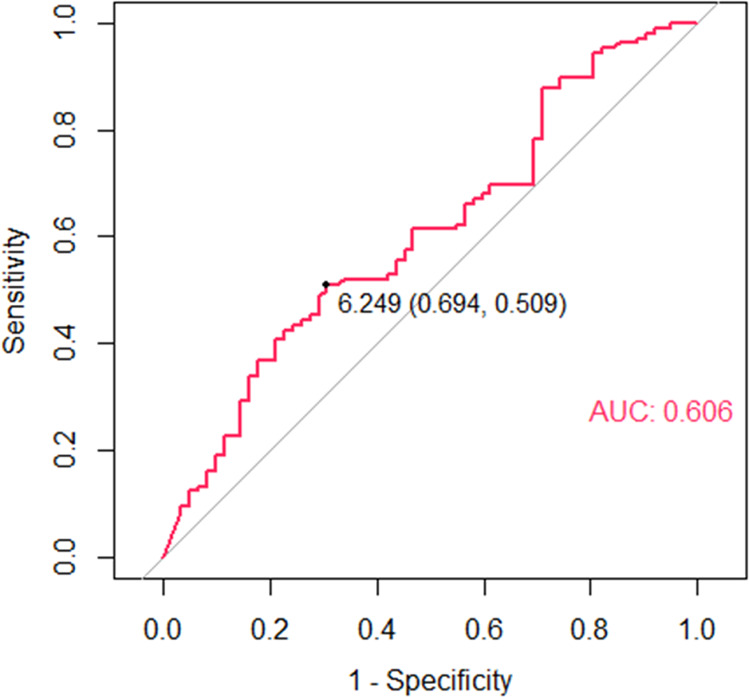
Receiver operating characteristic (ROC) curve analysis of the cut-off value of VitB12 level predictive of insomnia. VitB12 levels are natural logarithm transformed.

To determine the VitB12 level predictive efficiency and cut-off value for predicting insomnia, we performed a ROC curve analysis. In particular, a VitB12 level >517.50 pg/ml (*P* = 0.01, AUC 0.61, standard error 0.04) was found to predict increased insomnia risk (Fig. 1).

Notably, we found that intake of mecobalamin (an active form of VitB12) was significantly more common in patients with insomnia. Moreover, correlation analysis indicated that serum VitB12 level was closely related to mecobalamin use (*r* = 0.40, *P* < 0.01). Notably, mecobalamin use was significantly more common among participants with DPN (20.9%) than those without DPN (3.1%) (*P* < 0.01).

## Discussion

In the present study, we evaluated the associations between micronutrients and the incidence of insomnia in patients with T2DM. We observed an insomnia prevalence of 24.16% in T2DM patients, which was similar to that reported by another large community-based Chinese study (20.2%). In that previous study (which enrolled 377 T2DM and 4741 nondiabetic controls), the prevalence of insomnia in T2DM patients was significantly higher than in nondiabetic controls, in which the prevalence of insomnia was only 12.2% [2]. In contrast to the results of some previous studies, our participants with insomnia included fewer alcohol drinkers compared to participants without insomnia. This could be explained by the greater number of women among the insomniac group, as Chinese women are much less likely to drink alcohol. Although DPN reportedly contributes to insomnia in T2DM patients, the proportion of patients with DPN was not higher in the insomniac group and therefore not relevant to insomnia. However, patients enrolled in most related studies reported painful DPN resulting in sleep problems and more severe pain leading to worse sleep quality [11, 32]. The patients enrolled in our study reported minimal pain (pain score <4 points), which might be a reason for the lack of association between DPN and insomnia in our study.

Micronutrients can affect sleep patterns by altering the levels of neurotransmitters and the expression of circadian genes. We therefore investigated several important micronutrients, including Vit D, folic acid, VitB12, Ca, K, Mg, P, and Fe in T2DM patients with and without insomnia. Interestingly, we found that only VitB12 levels were significantly increased in the insomniac group and independently associated with the presence of insomnia after adjusting for confounding factors (which differed between the two groups and have been shown to impair sleep). Also of note, the serum VitB12 level exhibited significant moderate value in predicting insomnia risk (AUC = 0.61). Even though some micronutrients, such as Ca, Mg, Fe, folic acid, and Vit D, have been shown by some researchers to exert a protective effect in sleep [14, 15, 33, 34] while K reportedly has the opposite effect [35], our study did not find any associations between these micronutrients and insomnia. We speculate that this discrepancy could have resulted in part from race/ethnicity differences. For example, one multi-ethnic study showed a significant association between Vit D deficiency and shorter sleep duration in African Americans, whereas this association was not found in Chinese Americans [16].

Currently, the clinical evidence supporting a relationship between VitB12 and insomnia are very limited and mixed, with both positive and negative findings. Mayer et al. [21] reported an alertness-increasing effect of VitB12 supplementation, with a decrease in sleep duration. Data from the US National Health and Nutrition Examination Survey for 2459 adults also showed an independent inverse relationship between serum VitB12 concentration and sleep duration [15]. A very recent study consistently found a strong negative association between sleep quality and VitB12 intake in healthy adults [22]. In contrast, another recent study by Soysal et al. [23] found no significant difference between insomnia severity and serum VitB12 level. Moreover, a study of 87 young and middle-aged adults [36], as well as another study of 355 healthy young female students [37], found a positive association between VitB12 and sleep, with higher dietary intake of VitB12 associated with better sleep. Unfortunately, we cannot make any inference as to the possible reasons for these differences in observations due to the limitations of the cross-sectional study design. Nevertheless, potential biological mechanisms underlying the association of increased VitB12 with poor sleep quality have been documented. Increasing levels of VitB12 in humans have been shown to lead to a decline in 24-h mean melatonin levels and nocturnal melatonin levels during bright-light exposure, thereby disrupting the sleep-wake cycle toward increased alertness and decreased sleep duration [21, 38]. Some rodent studies confirmed that VitB12 increases alertness to light stimulation; VitB12 phase-advances the circadian rhythm by increasing the light sensitivity of the circadian clock [39, 40]. Conversely, severe VitB12 deficiency reportedly causes hypersomnia, possibly due to a disruption in circadian rhythm [41, 42].

Interestingly, an inverse association between VitB12 and sleep was observed in the general population [15, 21, 22] and elder population (our T2DM patients), while this association was found to be reversed in the young population in two other studies [36, 37]. At present, we cannot explain this phenomenon. It is possible that VitB12 impacts sleep differently in older and younger adults, as the structure of sleep changes marked with aging. The circadian phase, response to light, and clock gene expression have been shown to differ between older and younger adults [43], which might cause a different response to VitB12.

Furthermore, we found that increased VitB12 levels in our participants may be at least, in part, relevant to the use of mecobalamin, which is used by T2DM patients in our department primarily for treating DPN. One study involving 20 healthy subjects showed that mecobalamin treatment for 14 days reduced sleep duration [21]. Whether long-term treatment with mecobalamin in T2DM patients with DPN harms sleep should be assessed in a future longitudinal study.

Some limitations of this study should be mentioned. First, insomnia was determined subjectively using self-reported sleep measures, which may be affected by estimation bias compared to more-objective measures of sleep, such as actigraphy and polysomnography. Second, although we adjusted for various confounding factors (age, sex, diabetes duration, alcohol use, Hb, and DPN), potential factors that could have a confounding effect remained, such as race/ethnicity, stress, and dietary habits. Thus, a larger-scale multi-center study will be considered in the future to minimize the potential interference of these factors. Third, the discrimination power of our VitB12 cut-off value was moderate. The accuracy of the predicting values also needs to be validated in a future study involving a larger population. Finally, due to the nature of cross-sectional study designs, we cannot interpret the causality of the observed association between VitB12 and insomnia. It would be interesting to unravel the sleep-related mechanisms regulated by VitB12 in future laboratory experiments.

In conclusion, our study showed that insomnia was prevalent in patients with T2DM. Increased serum VitB12 level was identified as an independent risk factor for insomnia and predicted increased insomnia risk. Our study adds more evidence supporting the inverse association between VitB12 and sleep. Longitudinal studies are needed to further investigate the cause-effect relationship between VitB12 and insomnia and establish a safe and adequate VitB12 supplementation level in patients with T2DM. Our findings in the Chinese patient population may or may not be generalizable to the world at large.

## Supplementary information

Supplemental table

## Data Availability

The datasets generated during and/or analyzed during the current study are available from the corresponding author (e-mail: geqian@hospital.cqmu.edu.cn) on reasonable request.
