# Supplementary material for: The independent association between vitamin B12 and insomnia in Chinese patients with type 2 diabetes mellitus: a cross-sectional study
Source: Nutr Diabetes. 2022 Jan 17;12:3. doi: 10.1038/s41387-022-00181-8 (PMC8764026; doi:10.1038/s41387-022-00181-8)
Supplement: Supplementary file 1 — Supplemental table [file 41387_2022_181_MOESM1_ESM.doc]

| Variables | Sensitivity | Variables | Sensitivity |
| --- | --- | --- | --- |
| C-peptide | 0.010 ng/mL | 25(OH)-Vitamin D | 4.0 ng/mL |
| HbA_1_C (%) | 3.8 | Cr | 5umol/L(0.06mg/dL) |
| TC | 0.1mmol/L(3.86mg/dL) | UA | 0.2mg/dL(11.9umol/L) |
| TG | 0.1mmol/L(8.85mg/dL) | Hb | 0 g |
| HDL | 0.08mmol/L(3.09mg/dL) | Potassium | 1.5(mmol/L) |
| LDL | 0.10mmol/L(3.87mg/dL) | Iron | 0.9umol/L(5.00ug/dL,0.05mg/L) |
| ALT | 5U/L(0.08ukat/L) | Magnesium | 0.10mmol/L(0.243mg/dL) |
| AST | 5U/L(0.08ukat/L) | Calcium | 0.2mmol/L(0.8mg/dL) |
| Phosphorous | 0.1mmol/L(0.31mg/dL) | Vitamin B12 | 50 pg/mL |
| Sodium | 80mmol/L | FT3 | 0.88 pg/mL |
| Folic acid | 2.0 ng/mL | FT4 | 0.25 ng/dL |
| Cortisol | 11 nmol/L | TSH | 0.01 uIU/mL |
| ACTH | 1.0 pg/mL |  |  |

Supplementary Table. The sensitivity of detection methods for biochemical parameters

Abbreviations: HbA1C=Hemoglobin A1c, TC=Total cholesterol, TG=triglycerides, HDL= high-density lipoprotein cholesterol, LDL=low-density lipoprotein cholesterol, ALT= Glutamic-pyruvictransaminase, AST=Glutamicoxalacetictransaminase, Cr=Creatinine and UA= Uric acid, Hb=Hemoglobin.
